# Supplementary material for: The genome sequence of the Spanish Festoon, Zerynthia rumina (Linnaeus, 1758) (Lepidoptera: Papilionidae)
Source: Wellcome Open Res. 2026 Jan 16;11:33. [Version 1] doi: 10.12688/wellcomeopenres.25724.1 (PMC12946699; doi:10.12688/wellcomeopenres.25724.1)
Supplement: Peer review history

# Reviewer report: Reviewer response for version 1

**DOI:** 10.21956/wellcomeopenres.28322.r146952

**Published:** 2026-02-26

**Authors:** Yoshiaki Morino

**Affiliations:** 1 University of Tsukuba, Tsukuba, Japan

A high-quality reference genome assembly is presented for the Spanish festoon butterfly *Zerynthia rumina*, generated from a female collected in Catalonia, Spain, using PacBio Revio HiFi and Hi-C data. The assembly was produced as a phased diploid assembly with two haplotypes using Hi-C–based phasing: haplotype 1 comprises 852.84 Mb, with 99.64% assigned to 31 chromosome-scale pseudomolecules, while haplotype 2 is provided at the scaffold level. A complete mitochondrial genome is also included. Data deposition and methodological reporting are thorough, with clear documentation of the software, pipelines, and manual curation steps, supporting transparency and reproducibility. Assembly quality metrics are strong, including high BUSCO completeness (lepidoptera_odb10; 99.0% complete for haplotype 1), and the reported EBP summary metrics meet recommended standards. Overall, this resource should be highly valuable for downstream studies in evolutionary biology and taxonomy.

Are sufficient details of methods and materials provided to allow replication by others?

Yes

Is the rationale for creating the dataset(s) clearly described?

Yes

Are the datasets clearly presented in a useable and accessible format?

Yes

Are the protocols appropriate and is the work technically sound?

Yes

Reviewer Expertise:

Evolutionary developmental biology, Invertebrate

I confirm that I have read this submission and believe that I have an appropriate level of expertise to confirm that it is of an acceptable scientific standard.

# Reviewer report: Reviewer response for version 1

**DOI:** 10.21956/wellcomeopenres.28322.r145111

**Published:** 2026-02-03

**Authors:** Maria Nilsson

**Affiliations:** 1 Senckenberg Biodiversity and Climate Research Centre, Frankfurt am Main, Germany

The nuclear genome has been sequenced from a butterfly, the Spanish festoon Zerynthia rumina.

 The species occur in the Mediterranean region, and the sample originate from Spain.

 The assembly is based on pacbio revio longread sequences and HiC. The final length of the manual curated assembly is 759Mb and contain 31 chromosomal pseudomolecules including both sex chromosomes. The assembly was done according to current best practice. All quality values and statistics is reported and indicate a high quality butterfly genome. The methodology and all tools and software used is reported. 

Are sufficient details of methods and materials provided to allow replication by others?

Yes

Is the rationale for creating the dataset(s) clearly described?

Yes

Are the datasets clearly presented in a useable and accessible format?

Yes

Are the protocols appropriate and is the work technically sound?

Yes

Reviewer Expertise:

phylogenomics, transposable elements, mitogenomics

I confirm that I have read this submission and believe that I have an appropriate level of expertise to confirm that it is of an acceptable scientific standard.
